# Embedding the remote sensing monitoring of archaeological site damage at the local level: Results from the “*Archaeological practice and heritage protection in the Kurdistan Region of Iraq*” project

**DOI:** 10.1371/journal.pone.0269796

**Published:** 2022-06-15

**Authors:** Elise Jakoby Laugier, Nawzad Abdullatif, Claudia Glatz

**Affiliations:** 1 Department of Anthropology, Rutgers University, New Brunswick, New Jersey, United States of America; 2 Center for Human Evolutionary Studies (CHES), Rutgers University, New Brunswick, New Jersey, United States of America; 3 Garmian Department of Antiquities, Kalar, Kurdistan Region, Iraq; 4 Archaeology, School of Humanities, University of Glasgow, Glasgow, United Kingdom; University of Liverpool, UNITED KINGDOM

## Abstract

Today, the satellite-based monitoring of archaeological sites and site damage is a widespread practice, especially in conflict-affected regions. However, the vast majority of these remote sensing cultural heritage monitoring efforts have been led and conducted by remote researchers, and there remains an urgent need to embed this work within existing, in-country institutions at local and regional levels. Here, we present the archaeological site monitoring approach and results from the project *Archaeological Practice and Heritage Protection in the Kurdistan Region of Iraq*, a collaborative project between the Sirwan Regional Project and Kurdish Iraqi archaeologists aimed at generating a fully functional and sustainable programme of archaeological site management co-created with, and managed by, Kurdish Iraqi archaeologists and antiquities officials. Between August 2018 and February 2020, 376 archaeological sites in the Sirwan/Upper Diyala River Valley region, located in the Kurdistan Region of Iraq, were assessed for damage by Kurdish Iraqi archaeologists in collaboration with the Sirwan Regional Project. This work represents the first large-scale, systematic dataset of archaeological site conditions and longer-term damage in the Kurdistan Region of Iraq (KRI). Our results show that 86.7% of the assessed archaeological sites and 38.6% of the site surface area in this region were affected by damage between 1951–2018, and demonstrate the great urgency with which action must be taken to develop appropriate safeguarding measures for the KRI’s archaeological heritage. On the basis of these results, we outline relevant recommendations for the immediate protection of archaeological sites in Garmian and the greater Kurdistan Region.

## Introduction

The remote monitoring of archaeological sites and site damage using satellite imagery represents today a widespread practice, especially in regions affected by conflict [1, 2]. Most day-to-day efforts of cultural heritage protection work are conducted by national and regional departments of antiquities. Yet, the majority of remote sensing-based archaeological monitoring projects for Western Asia are primarily led, carried out, and publicised by researchers based in Europe and North America (e.g., [3–5]). However, the sustainable and ethical management of cultural heritage requires the integration of local knowledge, expertise, and leadership at all levels [6–8], including the use and analysis of advanced geospatial data [9, 10]. The project *Archaeological Practice and Heritage Protection in the Kurdistan Region of Iraq* (https://culturalheritageprotection.org) addressed the urgent need for the development of not only Iraqi experts in archaeological site monitoring and damage assessment, but also the necessity of embedding this work within existing, in-country institutions at local and regional levels that are tasked with the protection and management of cultural heritage [11]. The project was co-developed and co-led by a partnership between the University of Glasgow, the Institute for Heritage and Sustainable Human Development (Inherit), and the Suleymaniyah Department of Antiquities in collaboration with the Garmian Department of Antiquities, the Garmian Civilization Museum, the Slemani Museum, and the Kurdistan Regional Government’s (KRG) General Directorate of Antiquities in Erbil.

To create a broad public base for the protection and sustainable management of cultural heritage in the Kurdistan Region of Iraq, *Archaeological Practice and Heritage Protection in the Kurdistan Region of Iraq* co-created with local partners two new museum spaces in Kalar and Suleymaniyah, a series of school education kits, and rural engagement programmes [11, 12]. These provide children, young people, and a wider public with the opportunity to engage with archaeology as a practice, and with archaeological heritage as an important means of understanding the local past. The project also co-designed a system to digitally and sustainably monitor and protect archaeological sites in the region.

The aim of the site monitoring component of the *Archaeological Practice and Heritage Protection in the Kurdistan Region of Iraq* project has been to co-create a fully functional and sustainable programme of archaeological site management co-created with, and managed by, Kurdish Iraqi archaeologists and antiquities officials. Unlike previous projects, the training and archaeological site monitoring presented here is embedded within this wider, holistic, and forward-looking approach to cultural heritage that is concerned with the widening of access to, enjoyment of, and responsibility for cultural heritage and its safeguarding.

**Fig 1:**
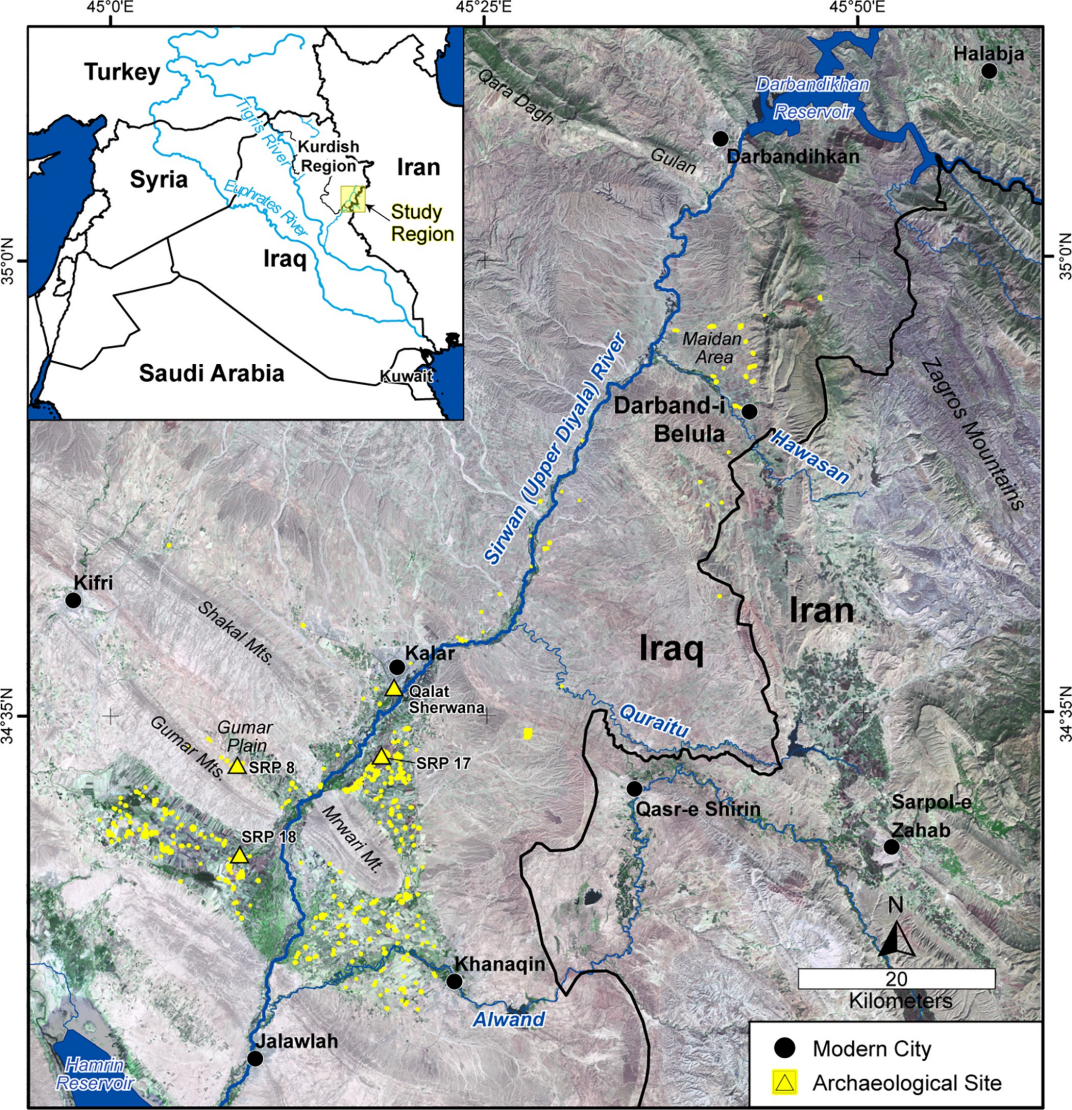
Map of the Sirwan/Upper Diyala River region in the Kurdistan Region of Iraq showing the location of sites monitored by the project (yellow). Archaeological sites highlighted in the text and figures are indicated as yellow triangles. The basemap is Landsat 8 natural color imagery (acquired 4 October 2021) freely downloaded from http://earthexplorer.usgs.gov (courtesy of the USGS and NASA).

The project’s archaeological site monitoring component developed out of the work of the Sirwan Regional Project (SRP) [13], a regional archaeological survey and excavation project located in the Garmian region of the Kurdistan Region of Iraq (KRI) [14–17] (Fig 1). Starting from August 2018, SRP researchers trained and subsequently supported a funded, full-time Cultural Heritage Monitoring Officer and other archaeologists working for the Garmian Directorate of Antiquities and the Garmian Civilization Museum to allow them to take over the satellite remote sensing and the field-based aspects of archaeological site monitoring and condition assessment, previously carried out by the SRP team. By iteratively monitoring sites from satellite imagery and visiting them in person, the Garmian archaeologists developed an expertise for identifying the range of damage types and threats impacting archaeological sites.

As a result of this work, every confirmed archaeological site in the Sirwan/Upper Diyala River Valley and adjacent areas has a digital record and damage assessment. A total of 376 sites (722.4 hectares, or ha) were assessed for damage between August 2018 and February 2020 resulting in 1307 individual assessments of damage. Of those 376 sites, 326 (86.7%) were found to have sustained some form of damage impacting 278.9 ha, or 38.6%, of the site surface area in the region. These results demonstrate the urgency with which action needs to be taken to develop culturally and socially sensitive safeguarding measures for the region’s and the wider KRI’s archaeological heritage.

In this paper, we (1) describe the workflow used to co-create and maintain the archaeological site and damage database, (2) analyse the monitoring data in more detail, and (3) make preliminary recommendations for the future safeguarding of archaeological sites accordingly.

## Materials and methods

The site monitoring component of the *Archaeological Practice and Heritage Protection in the Kurdistan Region of Iraq* project was conducted by members of the Garmian Department of Antiquities, the Garmian Civilization Museum, and the Sirwan Regional Project (SRP). Permission for all fieldwork reported in this study was granted by the Kurdistan Regional Government’s (KRG) General Directorate of Antiquities in Erbil through archaeological permits issued to the Directorate of Antiquities and Heritage of Garmian and the Sirwan Regional Project (SRP). Additional information regarding the ethical, cultural, and scientific considerations specific to inclusivity in global research is included in S1 Checklist.

All necessary equipment and software to carry out the monitoring, recording, and visualisation in this study was purchased by the project *Archaeological Practice and Heritage Protection in the Kurdistan Region of Iraq* and donated to the Garmian Department of Antiquities to ensure the sustainability of local site monitoring and management beyond the lifetime of the project. Table 1 summarizes the imagery and historic photographs used in this study. High-resolution WorldView-2 satellite imagery was provided by the Sirwan Regional Project (SRP) for the initial training and database generation. Moving forward, we have begun transitioning to using open-source imagery (e.g., Bing, Google, etc.) for regional monitoring as it is available and drone images as superior quality imagery (< 5 cm/pixel) for performing damage assessments and temporal analyses (see section: *General Workflow*).

**Table 1:** The satellite imagery and historic aerial photographs used to assess archaeological sites in this study.

Data Set	Date	Spatial Resolution (m^2^)	Regional Coverage (No. of sites visible; Site area, ha)	Description
**Royal Airforce (RAF) Aerial Photography (HAS-P Series)**	Nov-1951	~2	205 (348.0)	Historic photographs
**CORONA KH-4B**	3-Aug-1969	1.8–2.7	376 (722.4)	Historic imagery (USGS; CORONA Atlas)
**WorldView-2 Imagery**	13-Feb-2011 20-Jan-2015 17-Aug-2018	0.46	375 (720.6) 329 (611.5) 327 (613.6)	High-resolution imagery (Maxar Technologies)

For historic imagery, the project leveraged historic 1951 Royal Airforce (RAF) HAS-P series aerial photographs and declassified CORONA spy satellite imagery from August 1969 downloaded from the CORONA Atlas (http://corona.cast.uark.edu) (1107–2170 Aft). Historic imagery is an invaluable resource, particularly in (semi-)arid Western Asia, for identifying and visualizing archaeological sites and their surrounding landscapes that have been obscured or destroyed by development and intensive agricultural activity over the last several decades [18–22].

### Archaeological site database

This study’s regional archaeological site database was created by the Sirwan Regional Project (SRP) and contains all known and potential archaeological site locations in the Sirwan/Upper Diyala valley and adjacent regions (Fig 1) [14–16]. The SRP survey approach involved first identifying and digitizing all potential archaeological sites using both historic CORONA and the 2011 Worldview-2 imagery and then systematically visiting each site in-person. Accordingly, the SRP database favoured the identification of sites that are highly visible on satellite imagery, such as sites with mounding, geometric edges, or sediment colour contrast. Flat sites, lithic scatters, and other site types with generally limited visibility on satellite imagery are likely underrepresented in the site database and in this study. While a site’s visibility in satellite imagery is an imperfect measure of occupational extent ([23, 24]; see also [16]), it is the most readily assessable means for quantifying both site and damage area. Thus, site areas, as they are used in this study, are primarily based on their visibility and extent of mounding in CORONA imagery, the earliest available imagery dataset at the time the SRP site database was created. Site damage status was determined using the earliest available imagery.

The archaeological site database is maintained in an ArcGIS geodatabase (v.10.6.1). Archaeological sites either have an “SRP” or “CPF” prefix and a unique identifying number (e.g., SRP 176). “SRP” sites are confirmed archaeological sites documented in-person by the SRP, and “CPF” sites are potential archaeological sites that have only been identified on satellite imagery. The majority of CPF sites are located to the south of the SRP research area (Fig 1). These sites were analysed as a comparative dataset using satellite and historic aerial imagery only; no ground-truthing was carried out at these sites as part of this project.

### Iterative damage assessments

#### General workflow

**Fig 2:**
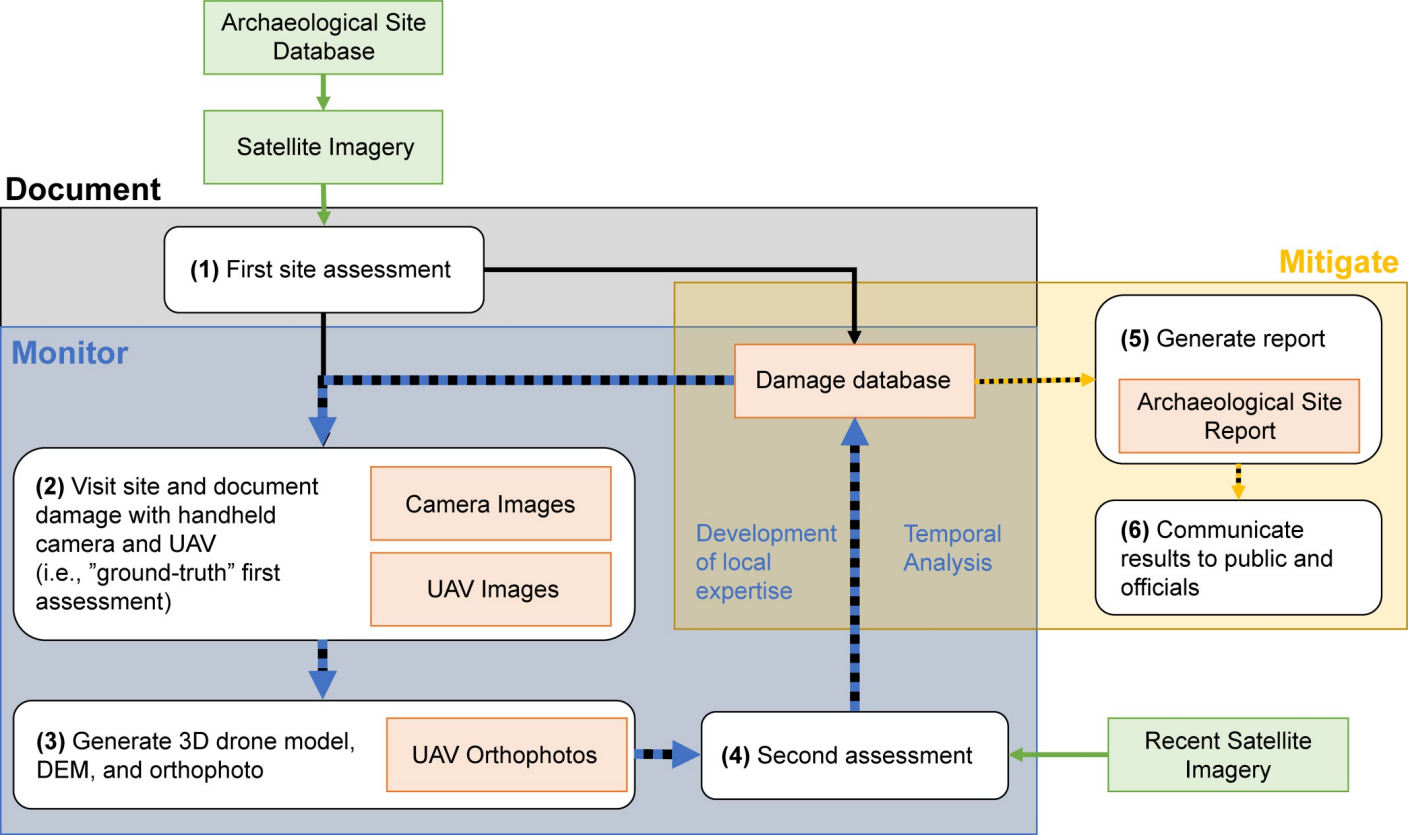
Graphic of monitoring objectives and workflow. The three main objectives are displayed in different colour semi-transparent boxes. Numbered white boxes indicate each workflow step. Green boxes are digital inputs to the workflow and orange boxes are digital data products. Dashed blue arrows specify the iterative processes in the workflow. Dashed yellow lines indicate site protection deliverables.

The main objectives of the site monitoring component of the *Archaeological Practice and Heritage Protection in the Kurdistan Region of Iraq* project were (A) to digitally *document* damage to archaeological sites, (B) to continually *monitor* sites for recent and impending damage threats, and (C) to produce preliminary recommendations for the development of measures that will allow stakeholders and policymakers to make informed choices about whether and how to *mitigate* damage to archaeological sites in the future (Fig 2).

**Fig 3:**
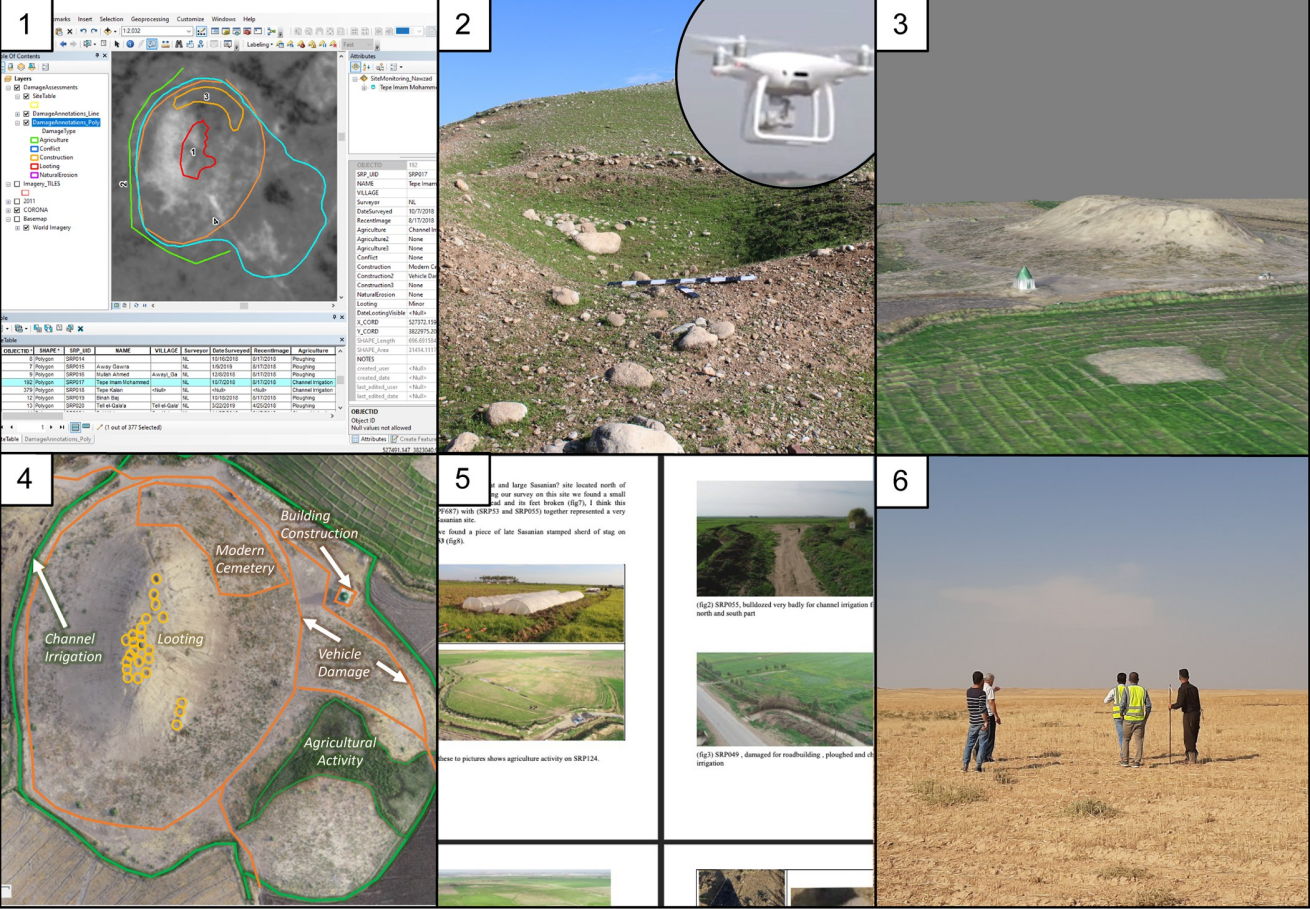
Walkthrough of workflow for a single site, Imam Mohammed (SPR 17). (1) Initial assessment of damage in ArcGIS software. The basemap is 1969 CORONA imagery (courtesy of the USGS and CORONA Atlas) overlaid with digitised damage assessments derived from the 2018 imagery. (2) Ground-truthing site visit and data collection. (3) Processing UAV data to generate a 3D model and high-resolution orthophoto of the site. (4) Conducting second damage assessment using UAV orthophoto. (5) Generating site report. (6) Communicating results to community and government officials.

The documenting and monitoring objectives were accomplished using an iterative six-step workflow: (1) initial assessment, (2) site visits and data collection, (3) data processing, (4) revised assessment, (5) generating site reports, and (6) site protection and public communication (Figs 2 and 3).

1. First, as part of the training process, SRP and Kurdish Iraqi researchers made an initial damage assessment of every site in the GIS database using 2018 Worldview-2 satellite imagery. This initial assessment was used to establish a baseline indicating the numbers of damaged sites in the region and train Kurdish Iraqi archaeologists to recognise archaeological site damage in satellite imagery.
2. Second, following this initial assessment exercise, Kurdish Iraqi archaeologists visited archaeological sites and took detailed pictures of individual instances of damage using both an unpiloted aerial vehicle (UAV, or drone) and hand-held cameras (Canon EOS 2000). Site visits were in effect “ground-truthing” the initial satellite-based assessments. They also systematically documented the entire site with a drone and **(3)** used the drone images to generate 3D models, digital elevation models (DEMs), and topographically-corrected image mosaics, or orthophotos, of the site (see the *Timing of damage* section for more detail).
4. Fourth, using the camera photos and high-resolution orthophotos from the site visits, they performed a second damage assessment, updating the initial damage assessments to accurately reflect a site’s damage status. The differences between remote and in-person assessments gave surveyors an exceptional idea of the levels of uncertainty, strengths, and weaknesses inherent in satellite-based (remote) damage assessments. Additionally, the project’s GIS damage database allows assessments to be queried geographically, temporally, and by damage type.
5. Fifth, they generated a detailed and easily accessible report of every site in the region. Together, the digital database, reports, and their experiences visiting sites have allowed the project team to isolate which archaeological sites are most threatened and what types of damage are most prevalent in the region.
6. Finally, Garmian archaeologists used their analysis to inform the local public, landowners, developers, and other government officials of the dangers posed to archaeological sites, the cost of cultural heritage losses, and how they can work together to minimise further damage.

### Photogrammetric models and orthophotos

As part of step 3 in the workflow, project surveyors produced photogrammetric models based on drone images of every site that was physically accessible between August 2018 and February 2020 in order to create an archival record of archaeological sites in the region and their condition. Automated drone surveys were planned in the Pix4DCapture App on an iPhone 6 and deployed with a DJI Phantom 4 Pro. Flights were generally flown at altitudes less than 36 m above ground level to achieve sub-centimetre resolution, or ground sampling distance (cm/pixel). Ground control points (GCPs) were collected using the EMLID Reach RS+ single-band RTK GNSS system or manually chosen from fixed points in available satellite imagery. Drone images were processed in Agisoft Photoscan (2018) and Metashape (2019–2020) software on an HP ZBook Core i7. 3D models were exported in universal.obj format and orthophotos were exported as.tiff files. Orthophotos were used for digitizing damage assessments in ArcGIS, thereby integrating the GIS and photogrammetry portions of the project.

### Damage types and severity

Damage to archaeological sites was digitized as polygons in ArcGIS on relevant satellite images or drone-acquired orthoimages. The damage and site database files were linked based on site ID (e.g., SRP 46) using the related tables function within the ArcGIS file geodatabase. While the database and analyses were conducted in ArcGIS, data and results are fully compatible with free GIS software packages, such as QGIS.

Five main categories of damage were recorded: agriculture, conflict, construction, natural erosion, and looting. Sub-types of each damage category are listed in S1 Table. Damage types are based on classifications used by the Endangered Archaeology in the Middle East and North Africa (EAMENA) project (http://eamena.arch.ox.ac.uk/), which is led by the Universities of Oxford, Leicester, and Durham, [3, 25] and the American Schools for Oriental Research’s Cultural Heritage Initiatives (e.g., [4, 26]). Importantly, the damage database used in this study represents a snapshot of the progress made by the Garmian archaeologists between August 2018 and February 2020. The damage database is an important part of the practice and protection of cultural heritage in the region, and the local Antiquities Department has continued to use and update the database since February 2020.

In this study, we assessed the impact of damage to archaeological sites in two ways: the number of sites damaged and the surface area in hectares affected by each damage type. When expressed as a percentage, the number of sites damaged captures the *ubiquity* of a type of damage across the region while the area affected provides an indication of the overall *spatial impact* of each damage type. All percentages are calculated out of 376 sites and 722.4 ha. The total site surface area damaged between 1951–2018 and for each damage category was calculated using the spatial “merge” function in ArcGIS. Repeated damage to the same site or area is captured by the temporal analysis discussed in the next section.

In many ways, defining and assessing the “severity” of damage to archaeological sites is an ongoing challenge for remote sensing cultural heritage specialists. While neither the number of damaged sites nor the area affected by damage is a perfect proxy for damage severity, taken together, these metrics capture two dimensions of “severity” that are immediately useful for defining and addressing the threats to cultural heritage in the region and can be built upon in future studies. The strengths and limitations of these proxies will be addressed throughout this study.

### Temporal analysis of damage

SRP researchers and Garmian archaeologists also jointly conducted an analysis of the timing of site damage using five sets of satellite or historic aerial imagery dating to between 1951 and 2018 (Table 1). As part of the project’s monitoring workflow, surveyors assessed the visibility of every site assessment by marking damage as “Visible” or “Not Visible” on each set of imagery (reported as “Yes” and “No” in S2 Table). The visibility of new damage allowed surveyors to establish the approximate time frame for when damage episodes occurred. For example, damage visible in both 2015 and 2018 imagery but not in 2011 imagery likely occurred in the four years between February 2011 and January 2015. Newer damage noted during site visits (i.e., occurring in late 2019–early 2020) was not included in this study’s temporal analysis.

Notably, imagery availability and the ability to perform a damage assessment differed in each time step. Unlike the untimed assessments of damage which are all calculated as percentages of the 376 sites or 722.4 ha, timed results were normalised by the number of sites and site area visible in each imagery dataset (Table 1). For example, the 1951 aerial imagery only covers 205 sites, and so the percent of sites damaged in 1951 is calculated out of these 205 sites. We then used the normalised percent of damaged sites and the normalised percent of damaged areas divided by the years elapsed between each imagery dataset to calculate the rate, or average number, of sites and percent area damaged per year. The rate cannot be calculated for the first timestep. The average number of hectares damaged per year was calculated independent of imagery availability (i.e., hectares/years elapsed).

Note also that the number of times a site is newly damaged may exceed the total number of sites affected by a particular type of damage. For example, a site may be newly damaged by ploughing activity in both 2011 and 2018 imagery. Thus, this one site will have two datable damage records and will be counted as a damaged site in both years. Likewise, site areas for each time step may also slightly exceed the total amount of damaged area because new damage may sometimes overlie older damage. The total damaged site area was calculated for each damage type in each time step using the spatial “merge” function in ArcGIS. The cumulative metrics only count an instance of damage the first time it occurs.

## Results

**Fig 4:**
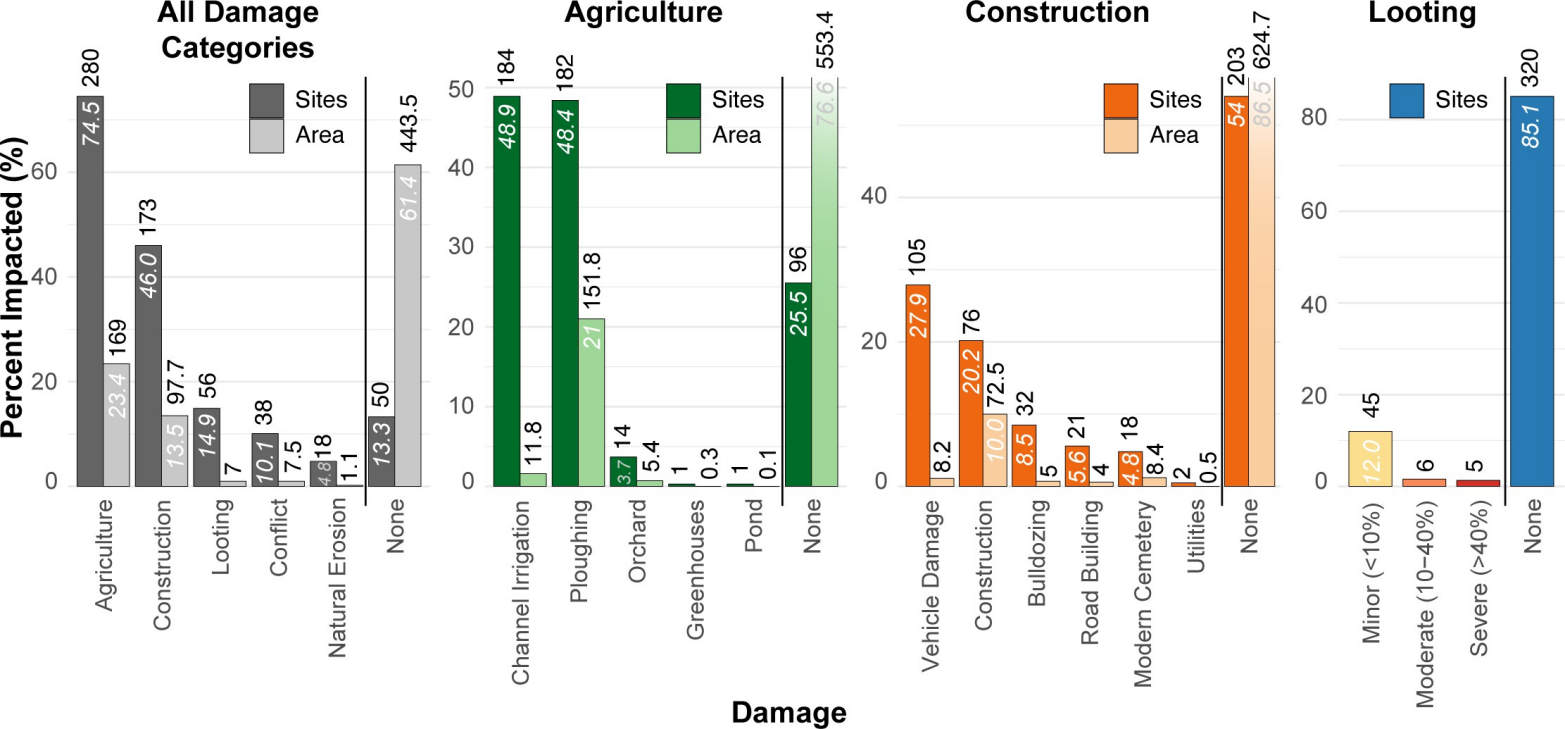
**Graphs showing the percentages of sites and site areas impacted by the major damage type categories (far left), the two most prevalent subcategories of damage, agriculture and construction (center), and looting (far right).** The percentages of sites and site areas (y-axis, labelled in italics inside bars) were calculated by dividing the number of sites and site area affected by each damage type (above bars) by the total number of sites assessed (376 sites) and the total site area (722.4 ha). Each site can be affected by multiple types of damage and thus summed percentages in each category may be greater than 100%.

**Fig 5:**
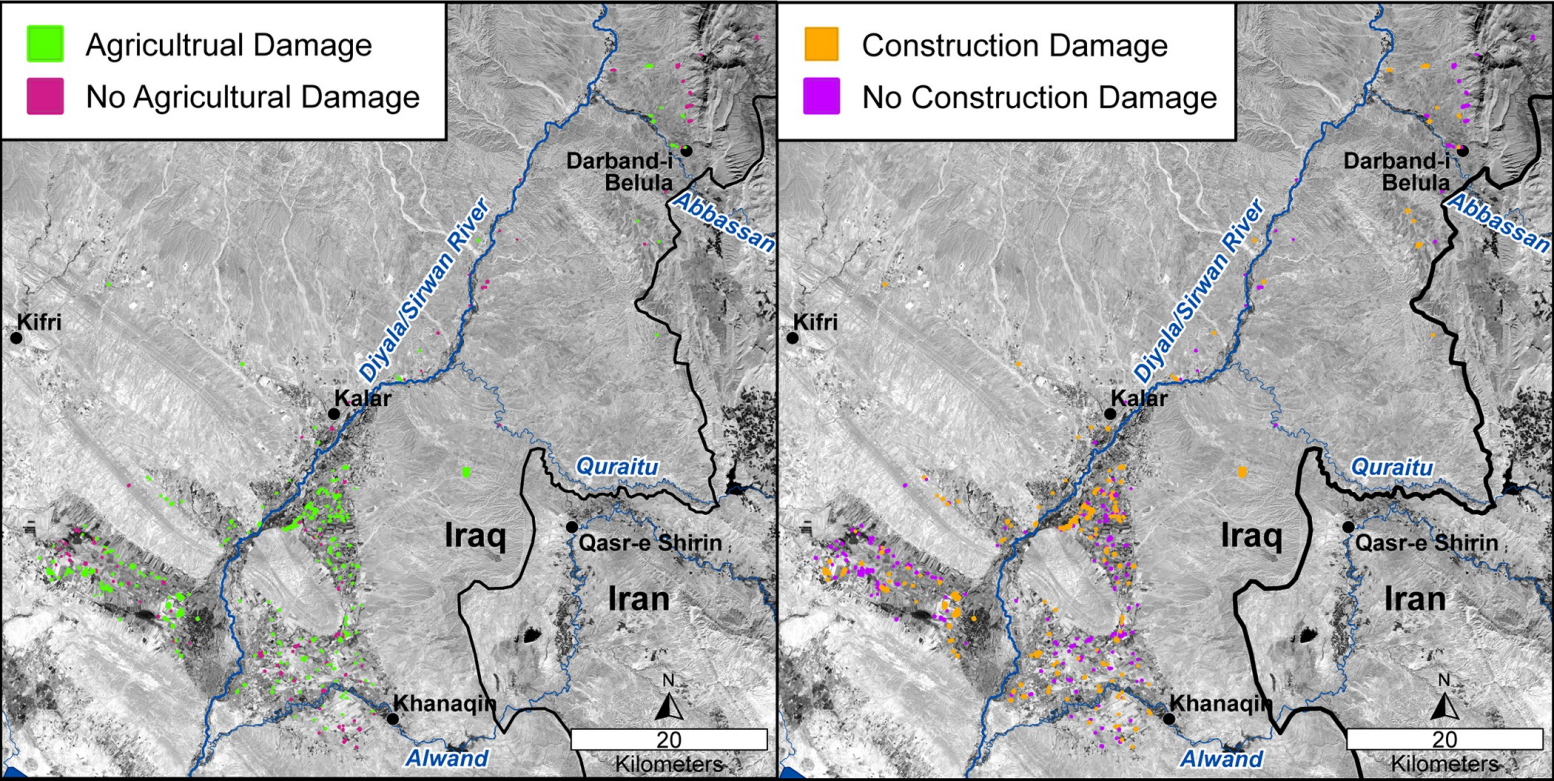
**Map of sites with agricultural (left) and construction (right) damage.** The basemap is Landsat 8 panchromatic imagery (acquired 4 October 2021) freely downloaded from http://earthexplorer.usgs.gov (courtesy of the USGS and NASA).

**Fig 6:**
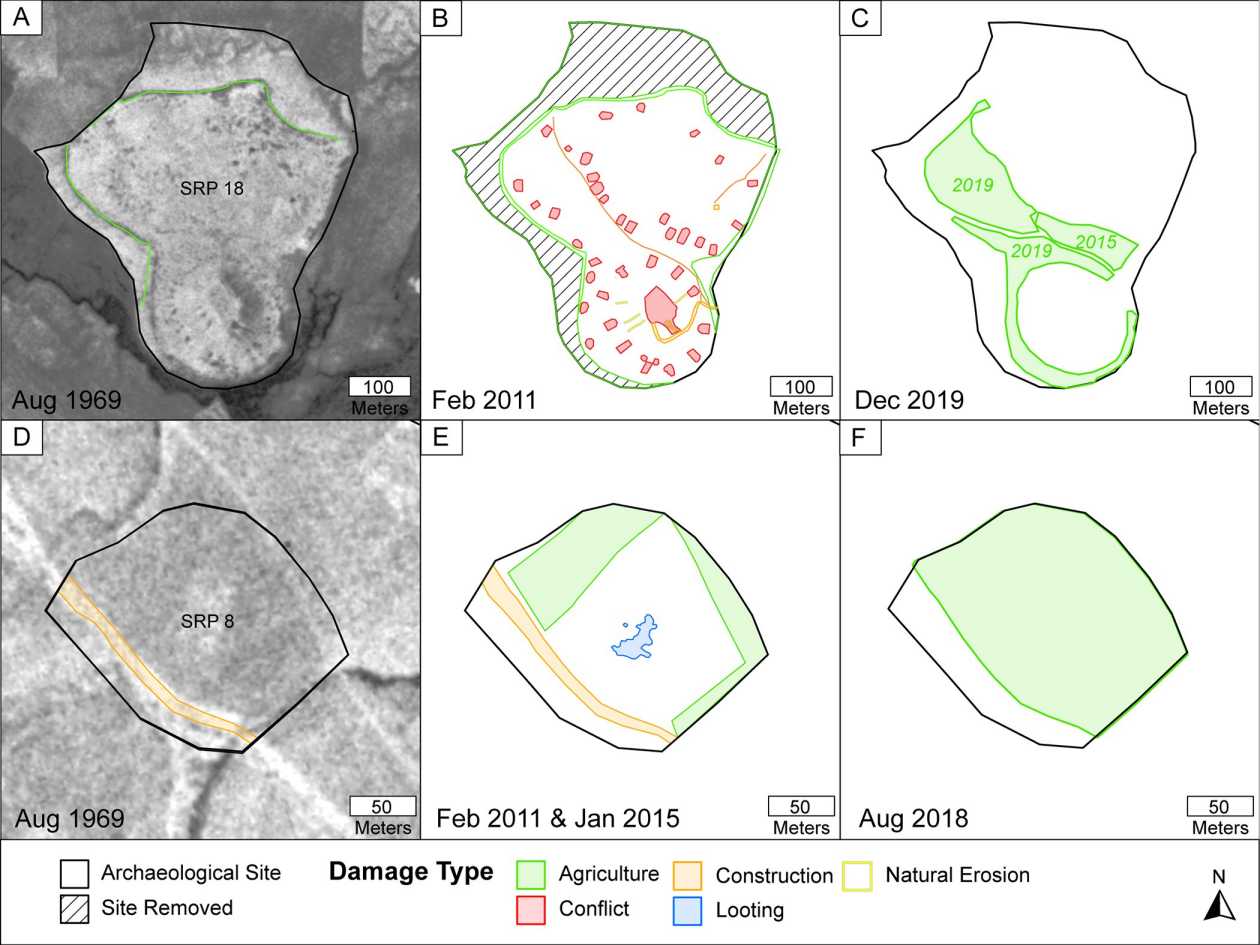
Damage assessments of Tepe Kalan (SRP 18; A–C) and SRP 8 (D–F). (A, D) August 1969 CORONA imagery (courtesy USGS and CORONA Atlas). See Fig 1 and CORONA Atlas URL links for site locations (SRP 18: https://corona.cast.uark.edu/atlas#zoom=17&center=5025288,4089876; SRP 8: https://corona.cast.uark.edu/atlas#zoom=18&center=5024420,4101263). (B–E) Damage assessments completed on 2011 Worldview-2 imagery. (C, F) Damage assessments reflecting post-2011 damage including new instances of agricultural ploughing that have obscured damage visible in earlier imagery (semi-transparent green areas). See Google Earth Pro (http://www.earth.google.com [accessed Feb 17, 2022]) for (C) December 2019, (E) January 2015, and (F) August 2018 imagery.

**Fig 7:**
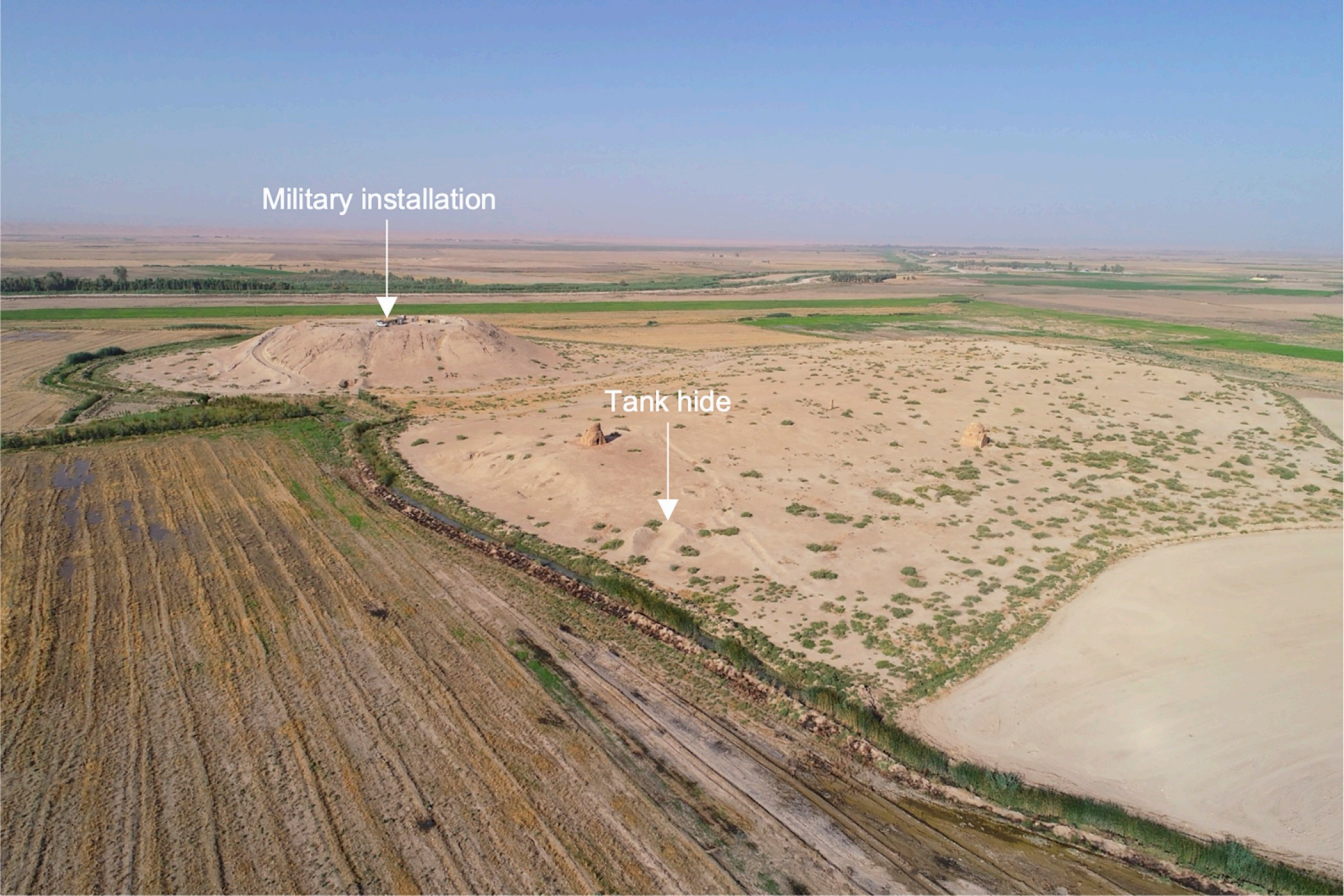
UAV-captured aerial view of Tepe Kalan (SRP 18) showing conflict-related damage (facing southwest). See Fig 1 for site location. Image courtesy of the Sirwan Regional Project.

**Fig 8:**
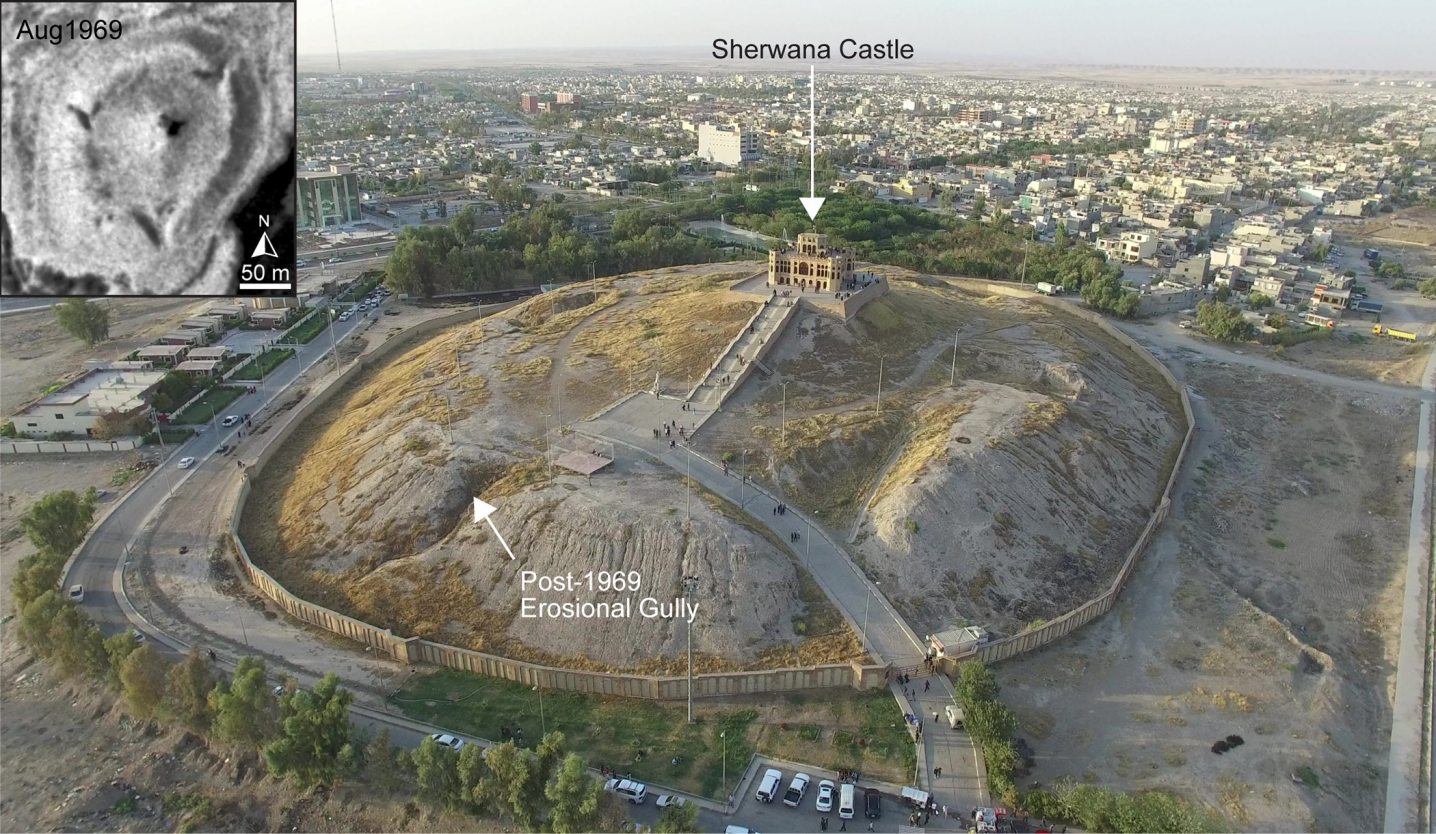
UAV-captured aerial view of Qala Shirwana showing erosional gullies on the sides of the mound (facing northwest). Figure inset shows the site as it appears in 1969 CORONA imagery (courtesy of the USGS and CORONA Atlas; https://corona.cast.uark.edu/atlas#zoom=16&center=5044266,4111020). See Fig 1 and CORONA Atlas URL link for site location. UAV image courtesy of the Sirwan Regional Project.

**Fig 9:**
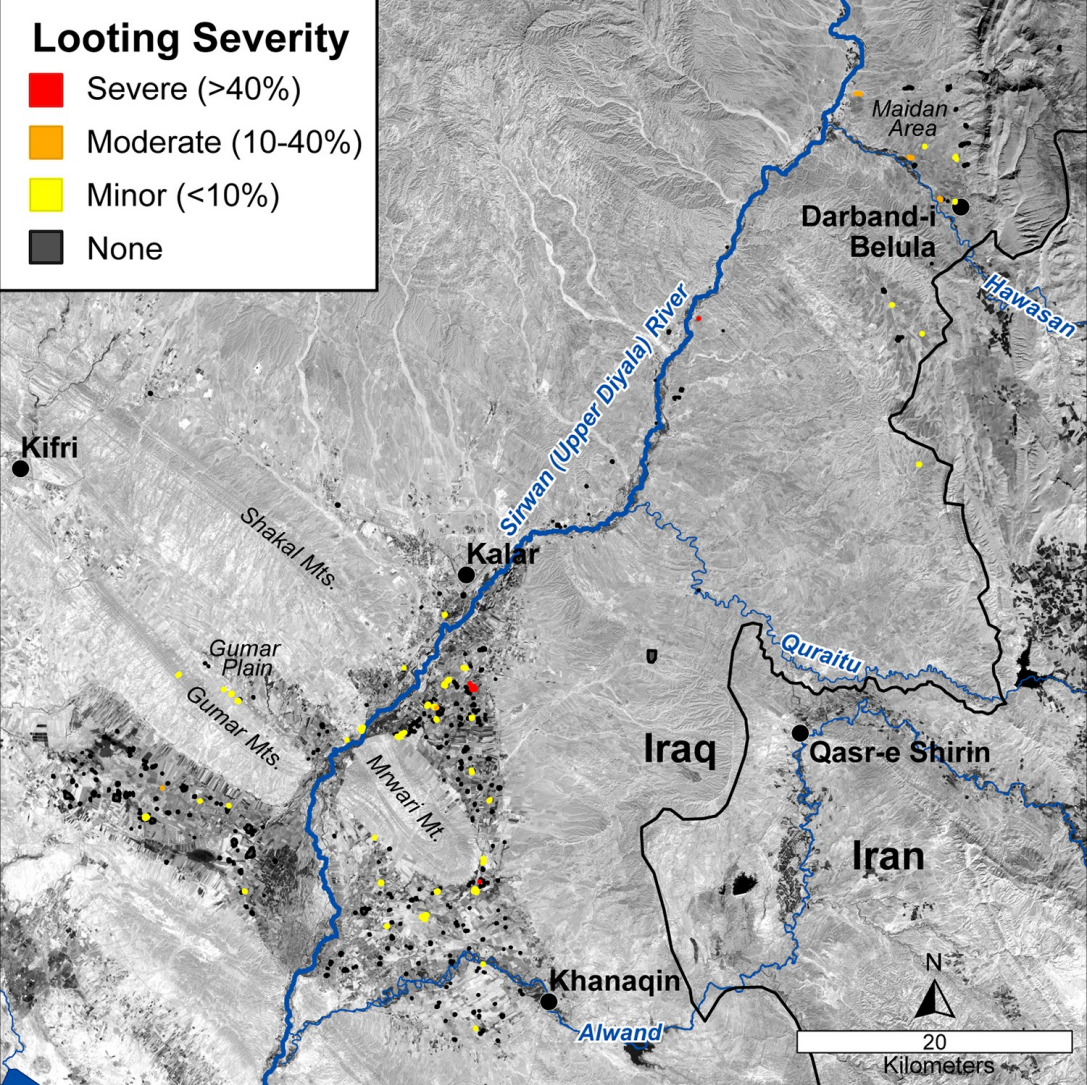
Map of sites affected by looting and coloured by looting severity (percent site area looted). The basemap is Landsat 8 panchromatic imagery (acquired 4 October 2021) freely downloaded from http://earthexplorer.usgs.gov (courtesy of the USGS and NASA).

Between August 2018 and February 2020, Kurdish Iraqi archaeologists, in collaboration with Sirwan Regional Project (SRP) researchers, assessed 376 archaeological sites for damage. Of those 376 sites, 326 (86.7%) were damaged in some way and 50 (13.3%) sites showed no signs of damage (Fig 4). The majority of damaged sites were usually impacted by multiple (sub-)categories of damage with a total of 1307 individual recorded instances of damage. It should be noted that site damage percentages overlap with multiple damage types and reflect the presence or absence of a damage type, not damage severity. Conversely, each damage assessment also provides a measure of the surface area impacted by each damage type. Of the 722.4 hectares of archaeological surface area in the region, 278.9 ha (38.6%) were damaged. S2 Table contains the final damage assessment database (2018–2020). Figs 4–9 present maps of site damage by category as well as detailed examples of satellite-based damage assessments.

### Damage types

#### Agriculture

The most common form of damage is the result of agricultural activity (280 sites, 74.5%) (Figs 4 and 5). 99 sites (26.3%) sustained two or more types of agricultural damage, and for 122 sites (32.4%), agricultural activity was the sole cause of damage (55.0 ha; 7.6%). In terms of site numbers, sites were nearly equally damaged by both ploughing and channel irrigation (Fig 4); however, ploughing impacted 151.8 ha of site area (21.0%), the most of any damage type, while irrigation canals only damaged 11.8 ha (1.6%) of archaeological site surface area in the region. In other words, irrigation damage is as ubiquitous as ploughing damage but causes much less surface area damage. This example highlights the importance of considering multiple proxies when assessing damage. Damage from orchards (14 sites; 3.7%) and greenhouses (1 site) were limited to a few archaeological sites in the region; although, tree planting and root activity are likely severely damaging to the underlying archaeology. No instances of damage related to agricultural terracing were recorded in the region.

The severity of agricultural damage is particularly difficult to quantify in this region and the impact from the same (sub-)categories of damage could range considerably. In seemingly minor cases of damage, sites were skirted by irrigation canals, leaving most of the site undamaged, or sites had clearly only been lightly ploughed. In more severe cases, large portions or even entire sites were completely obliterated by heavy ploughing and agriculture-related bulldozing. As an exceptionally severe example, Tepe Kalan (SRP 18) lost approximately 5.6 ha (~29%) of surface area to agricultural activity between 1969 and 2011 (Fig 6A and 6B). Regionally, for the seven years between 2017 and 2018, industrial-scale agricultural activity damaged 30.8 ha (17.8% of the total area impacted by agricultural activities) across the region (see the timing of damage section). However, we observed that damage was most severe where recent industrial-scale machinery was used to plough large areas of land or to dig new irrigation canals—an observation not fully captured by the surface area metric. Damage from irrigation is also more common and seemingly severe in sites farther south due to the decreased rainfall and increased need for irrigation infrastructure.

Thus, our results indicate that, on one hand, while we are likely not fully appreciating the severity of recent damage, on the other hand, *our results indicate that the long-term ubiquity and persistence of annual ploughing and canal maintenance are slowly and devastatingly removing archaeological sites*, *small portions at a time*. In many cases, agricultural activity has even begun to obscure other types of earlier damage, such as removing previous evidence for looting at SRP 8 (Fig 6E and 6F) and decades-old evidence of military activity at SRP 18 (Fig 6B and 6C).

#### Construction

Construction activities impacted 97.7 ha (13.5% of the total site area) at 173 sites (46.0%) making construction the second most damaging type of activity after agriculture (Figs 4 and 5). Persistent vehicle damage (105 sites; 29.7%) and building construction (76 sites; 20.2%) were the most common types of construction-related damage; but construction activities were much more damaging, impacting 72.5 ha (10.0% of total site area) while vehicle damage only impacted 82 ha (1.1%). Only 4 ha (0.6%) at 21 (5.6%) sites were damaged by road construction, but in these cases, the sites were almost always severely damaged and sometimes completely destroyed. While floodwaters from the Darbandikhan and Hamrin Dam constructions (completed in 1961 and 1981, respectively) have inundated numerous archaeological sites along the Sirwan/Upper Diyala River (Fig 1) [27, 28], no sites considered in this study were affected by dam construction or associated floodwaters. Like agricultural damage, construction-related damage is most pernicious in its persistence. The everyday activities of people driving to and from their homes over archaeological sites (especially in the wet season) as well as the slow expansion of towns and villages onto archaeological sites threaten the sites’ integrity. In most cases, it seems that people are aware that construction activities and continuous vehicular traffic can damage archaeological sites but not the extent or accretional style of damage caused by these activities.

#### Conflict-related damage

7.5 ha (1.0%) at 38 sites (10.1%) are damaged by conflict-related activities. This includes bulldozing for tank-hides, the creation of defensive berms, and military installations constructed on the tops of high mounds (e.g., Tepe Kalan (SRP 18), Figs 6A–6C and 7). All conflict-related damage occurred prior to our 2011 imagery and most likely occurred during the 1980s Iran-Iraq War. No instances of recent conflict-related deliberate damage (e.g. [29–31]) were recorded in the region. While no new instances of conflict damage were reported in the region, the damage is almost always severe and lasting.

#### Natural erosion

Only 1.1 ha (0.2%) at 18 sites (4.8%) are impacted by natural erosion, indicating that this type of damage is generally not a problem in the study region except at the largest mounds. Only a few known sites are threatened by flooding from the Hawasan and Sirwan/Upper Diyala Rivers. The deep erosional gullies on Qala Shirwana are a function of the mound’s size, morphology, and runoff from the 19^th^ century castle located at the top (Fig 8). Erosional gullies are also visible in the high mound of Tepe Kalan (Fig 6A–6C) and are also likely exacerbated by continued human activity on top of the site. As sites are composed mainly of mudbrick, similar types of erosion plague large archaeological sites across semi-arid Western Asia and are part of the natural decay of ancient settlements.

#### Looting

Fifty-six (14.9%) sites in this study showed signs of looting (Figs 4 and 9), but the vast majority of looting occurred prior to 2011 (because it is visible in the 2011 imagery). In terms of looting severity, 45 sites had only a few looting holes (<10% of each site area) and 5 sites were severely looted (>40% of site area; SRP011, SRP012, SRP013, SRP158, and SRP168). Locationally, looting appears to be more prominent in the northern part of the study area (Fig 9), but this is likely related to the larger population density. Within the higher population areas, looting tended to (but not exclusively) occur in more remote areas such as in the Gumar plain and northern portions of the Maidan area (Fig 1), or in conjunction with old military-related installations. Interestingly, many sites have been ploughed in a way that obscures earlier evidence of looting (e.g., SRP 8, Fig 6D and 6E). However, overall, looting seems to be a minor problem compared to other types of damage.

### Timing of damage

**Fig 10:**
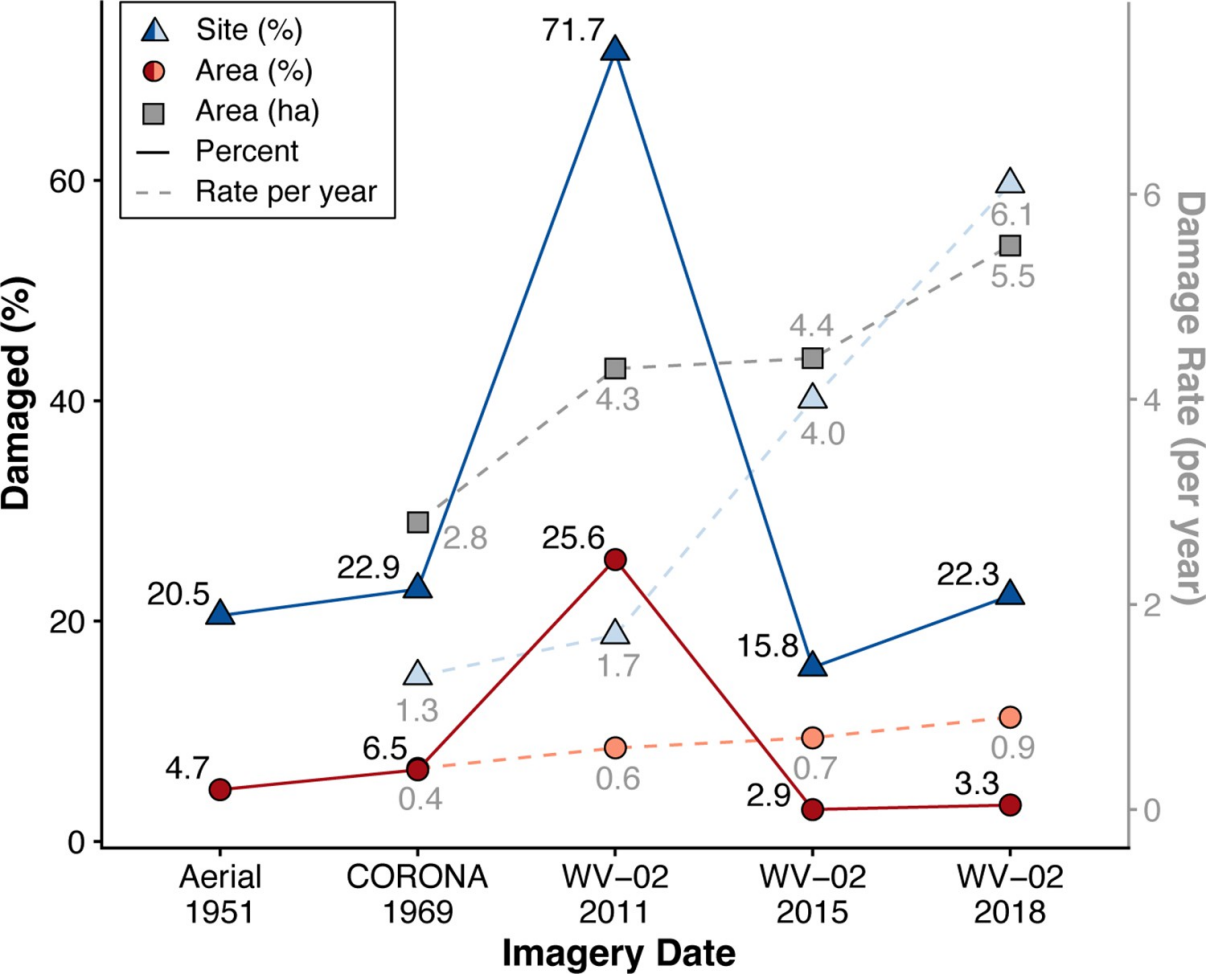
Graph showing the timing and rate of archaeological site damage between 1951 and 2018. The x-axis shows the date and type of the imagery. The left y-axis indicates the percentage (solid lines) of newly damaged sites (triangle) and site areas (circles) in each time step. The right y-axis indicates the rate of new damage per year based on the years elapsed between each new imagery dataset, including the percent sites (triangles), percent area (circles), and the number of new hectares (squares) damaged per year.

The temporal analysis of damage demonstrates that the vast majority of damage to archaeological sites in the study region occurred prior to 2011 (84.6% of the 1307 damage events, 78.7% of the 326 damaged sites, and 87.7% of the 278.9 ha of damaged area; Table 2, Fig 10; S2 Table). In fact, even in the earliest 1951 imagery, sites were subject to damage, particularly irrigation canals and buildings. Only 201 of the 1307 instances of damage were observed in the 2015 and 2018 imagery (reflecting the 7 years elapsed between 2011 and 2018). Damage during this time impacted 37.7 ha (13.5%) including 3.3 ha of which had been previously damaged. Damage occurred at 106 individual sites, 76 sites of which had previously been damaged. Of the post-2011 damage, agriculture and construction were the most prevalent types accounting for 37.0 ha, or 98% of new damage. Only 2.3% (0.2 ha) of looting took place post-2011 occurring at 8 sites. While less damage was documented between 2011 and 2015, all measures for the rates of new damage per year increased (Table 2; Fig 10).

**Table 2:** Archaeological site damage through time. The amount of newly damaged sites and site areas, percentages, and rates of damage for each imagery time step. The percentage values are italicised in parenthesis.*.

Imagery Time Step	Aerial 1951		CORONA 1969		WV-02 2011		WV-02 2015		WV-02 2018	
Damage Category	Area [ha (*%*)]	Sites [n (*%*)]	Area [ha (*%*)]	Sites [n (*%*)]	Area [ha (*%*)]	Sites [n (*%*)]	Area [ha (*%*)]	Sites [n (*%*)]	Area [ha (*%*)]	Sites [n (%)]
Agriculture	6.7 (*1*.*9*)	28 (*13*.*7*)	11.4 (*1*.*6*)	58 (*15*.*4*)	120.8 (*16*.*8*)	205 (*54*.*7*)	14.8 (*2*.*4*)	33 (*10*.*0*)	16.1 (*2*.*6*)	41 (*12*.*5*)
Construction	9.1 (*2*.*6*)	13 (*6*.*3*)	31.4 (*4*.*3*)	30 (*8*.*0)*	54.4 (*7*.*6*)	144 (*38*.*4*)	2.2 (*0*.*4*)	20 (*6*.*1*)	3.9 (*0*.*6*)	32 (*9*.*8*)
Looting	0.0 (*0*)	2 (*1*.*0*)	4.1 (*0*.*6*)	5 (*1*.*3*)	2.7 (*0*.*4*)	169 (*45*.*1*)	0.0 (*0*.*0*)	5 (*1*.*5*)	0.1 (*0*.*0*)	4 (*1*.*2*)
Conflict	–	–	0.0 (*0*.*0*)	1 (*0*.*3*)	7.0 (*1*.*0*)	36 (*9*.*6*)	0.5 (*0*.*1*)	3 (*0*.*9*)	–	–
Natural erosion	0.6 (*0*.*2*)	8 (*3*.*9*)	0.1 (*0*.*0*)	3 (*0*.*8*)	0.2 (*0*.*0*)	9 (*2*.*4*)	0.1 (*0*.*0*)	3 (*0*.*9*)	0.0 (*0*.*0*)	1 (*0*.*3*)
Total new damages *	16.5 (*4*.*7*)	42 (*20*.*5*)	47.0 (*6*.*5*)	86 (22.9)	184.4 (*25*.*6*)	269 (71.7)	17.6 (*2*.*9*)	52 (15.8)	20.1 (3.3)	73 (22.3)
**Cumulative damages**	16.5	42	63.5	120	244.5	296	260.8	309	278.9	326
**New damage events (n)**	59		117		930		80		121	
**Imagery years elapsed**	–		17		43		4		3.67	
*Rate of new damage* (% area/year)	–		0.4		0.6		0.7		0.9	
*Rate of new damage* (ha/year)	–		2.8		4.3		4.4		5.5	
*Rate of site damage* (sites/year)		–		1.3		1.7		4.0		6.1

*Note that a single site can be newly damaged in multiple time steps and by multiple types of damage in each time step, and thus the number and areas of newly damaged sites in each damage category may exceed the summed “total new damages” (e.g., in 2011, SRP 80 is counted as a newly damaged site in both the agricultural and construction damage categories, but SRP80 is only counted once in the “total new damages” row).

There are two important and related caveats to consider alongside these results: imagery resolution and return time. First, higher resolution imagery increases damage visibility and thus the amount of damage observed in the more recent imagery may be a function of its higher resolution. However, it should be noted that the 2011, 2015, and 2018 imagery all have the same resolution (pixel size) and thus are directly comparable. Conversely, in some instances, historic imagery was too low quality to make damage assessments. For example, damage by looting is generally not visible in CORONA imagery because hand-dug looting holes are often smaller than the imagery pixel size (c. 2 m). In fact, 145 of the 931 instances of damage first observed in the high-resolution 2011 Worldview-2 imagery (0.46 m) cannot be verified as present or absent in the preceding CORONA imagery due to its sometimes low-quality visibility. In this case, the two types of imagery are complimentary but may not be directly comparable.

Second, as reported in the looting results, we noted some instances where previous damage was obscured by new types of damage (e.g., looting holes visible in 2015 imagery were obscured by subsequent ploughing activity visible in 2018 imagery, Fig 6D and 6E). Thus, the short return time between the 2011, 2015, and 2018 imagery may have allowed surveyors to record more instances of damage than in previous time steps with longer durations. Despite these important caveats, we can tentatively say that the rate of damage to archaeological sites has increased over the past decade and results offer important insights for the management of cultural heritage in the region.

## Discussion

### An embedded, sustainable approach to cultural heritage management

With its focus on knowledge transfer and exchange, long-term collaboration, and the empowerment of local experts, the *Archaeological practice and heritage protection in the Kurdistan Region of Iraq* project represents a step-change from previous international, remote sensing-based cultural heritage monitoring initiatives, especially those conducted in times of crisis, conflict, and unrest (summarised by [32]). The project made concerted efforts not only to link the remote sensing and on the ground monitoring activities, but also to ensure that this linked approach was embedded within and managed by local archaeologists, agencies, and institutions with first-hand knowledge of archaeological sites and how best to manage their protection. In doing so, the project has taken practical steps toward the decolonisation and fulfilment of ethical obligations in cultural heritage management that have been advocated by archaeologists across the globe (e.g., [9, 10, 33–35]), and which still represents a particularly urgent endeavour for the Middle East [6].

Specific outcomes of this approach include (1) the first large-scale, systematic dataset of archaeological site conditions and longer-term damage in the Kurdistan Region of Iraq (KRI), (2) the co-development of cultural resource management expertise, and (3) a sustainable programme of cultural heritage management. (1) The co-created monitoring dataset, managed by local archaeological and cultural heritage professionals, now provides up-to-date and easy-to-digest information on the status of archaeological sites and their most immediate threats that can be directly relayed to stakeholders and regional and national heritage bodies and policymakers. (2) By iteratively monitoring sites from satellite imagery and visiting them in person, Kurdish Iraqi and SRP archaeologists co-developed expertise for identifying the range of damage types impacting archaeological sites in the study region both on the ground and in remotely sensed imagery. (3) The work is also sustainable in that the monitoring programme can function indefinitely without continued involvement from the SRP. Local archaeologists who have gained expertise in archaeological site monitoring and assessment can pass these skills on to colleagues and other KRI antiquities departments. Moreover, project members have begun to and will continue performing damage assessments and temporal analyses using open-access GIS software (i.e., QGIS), open-source satellite imagery (e.g., Bing, Google, etc.), and newly collected drone-derived orthophotos.

When considering sustainability, we do acknowledge the time and labour involved in cultural heritage work and recognise that ongoing efforts are supported by a local government currently experiencing a financial crisis. Thus, the financial sustainability of the monitoring programme may require new funding inputs, such as those offered by the Aliph Foundation or Arcadia Fund. Semi-automated or machine learning approaches are also showing promise for aiding cultural heritage research in Iraq and beyond (e.g., [36–40]), and these advanced approaches could potentially ease the time investment required by a manual approach to remote sensing-based monitoring. However, automated approaches not only require further technical development, they also need to engage the efforts and expertise of local antiquities departments before they can be considered as sustainable as the approach presented in this study.

### Current site threats and future monitoring work

The project’s analysis has provided critical insights into the status of archaeological cultural heritage in the region for the period between 1951–2018. Results demonstrate that 38.6% (278.9 ha) of site surface area from 86.7% (326) of sites in the region have been damaged, predominately by agricultural activity (169.0 ha, 23.4%; 280 sites, 74.5%) with nearly a third of sites (32.4%) reporting agriculture as the sole cause of damage. Conversely, looting overall appears to be a minor issue in the study region with only 1% of site area (7.0 ha) displaying looting-like damage across 56 sites (14.9%). Since 2011, looting has only been recorded at 8 sites (1.1%). These results align well with studies in the greater Middle East and North Africa (MENA) region and beyond reporting agriculture, urbanisation, and development as the most destructive and on-going threats to cultural heritage––not looting [26, 41–45].

For future research, we do see some room for improvement in the study’s temporal analysis that could provide more resolution to the long-term trends in archaeological site damage. In particular, future work could include additional, higher resolution imagery datasets between the 1950s and 2011. For example, 1950s–1960s U2 aerial imagery was collected at the same time as the CORONA imagery (1959–1972), but U2 images are much higher resolution and are varyingly available across populated areas of Western Asia [46]. For the period between 1971–1986, declassified, high resolution (< 1 m) HEXAGON KH-9 imagery is available across the globe [47–49] and provides an important time frame missing from this study. It should be noted that both U2 and HEXAGON imagery have either significant accessibility or spatial distortion challenges that currently require access to the National Archives and Records Administration (NARA) in the USA or georeferencing expertise to overcome [46, 49]. However, in the future, the development of online repositories and georeferencing tools like the CORONA Atlas and its associated “Sunspot” referencing system (https://sunspot.cast.uark.edu/) [22, 50, 51] will likely make U2 and HEXAGON historic imagery more readily accessible [49]. Finally, imagery from the IKONOS (0.80 m; 1999–2015) or Quickbird (0.65 m; 2001–2015) satellites captured before the 2003 US-led invasion of Iraq would also provide an important comparative time-step in the temporal analysis of damage to local cultural heritage (e.g., [45, 52]). Some of these imagery datasets are freely available, needing only to be tracked down and georeferenced, while others will require additional funding to acquire.

We also suggest that future studies make more concerted efforts to quantify the severity of all types of damage, but particularly agricultural damage. In this study, our two proxies for damage impact, the number of sites and the amount of surface area damaged, provided critical insight into the types and degrees of damage present in the region. However, there remains a range of nuance, such as the impact of different intensities of ploughing, that may only be evaluated through volumetric analyses. Change-over-time volumetric calculations using UAV-derived digital elevation models (DEMs) are widely used in other disciplines for erosional and crop monitoring (e.g., [53–56]). As part of this project, UAV-derived DEMs have already been generated for most sites in the Sirwan/Upper Diyala region and volumetric analysis will form an important part of the next phase of cultural heritage work in the region.

Relatedly, UAVs could also be useful for documenting and monitoring archaeological site types that were not considered in this study but are also likely to have been heavily damaged by agricultural activity: low-visibility flat sites (see *Methods* section). Flat sites and lithic scatters form an important part of the archaeological understanding and cultural heritage in the region [57], and ongoing and future work in the region is already making more concerted efforts to document these sites and their threats. Recent studies across the KRI have shown that UAVs and UAV-deployed sensors can be successfully used to document these otherwise difficult to detect sites and other associated archaeological features [17, 57–60].

As UAV-related technologies continue to rapidly improve and become more cost-effective, local antiquities departments may begin to use UAVs––as demonstrated in this study––as well as more advanced geospatial approaches as routine parts of an integrated cultural heritage monitoring approach. In either case, advanced UAV-derived analyses have great potential to improve both archaeological and cultural heritage endeavours, and going forward, the prospective use of these UAV approaches highlights the necessity of embedding cultural heritage approaches at the local level.

### Recommendations for local and regional cultural heritage protection

Based on the results of our monitoring efforts, we recommend the following steps be taken to reduce the number of archaeological sites being damaged in the future—and many of these recommendations have already begun to be implemented in the Sirwan/Upper Diyala region. As is the approach of cultural resource management (CRM) around the world, heritage protection cannot be treated in isolation from other social, political, and economic realities [8], and our recommendations reflect these realities. The recommendations are also specifically tailored to the study region. While similar empirical data is not yet available for other parts of the KRI and the rest of Iraq, the fact that our results align with studies across the greater Middle East and North Africa region suggests that our recommendations will be similarly applicable to the wider region.

#### Raising public awareness

A key step has to be the raising of public awareness, appreciation, and understanding of archaeological sites and the provision of information on how to report site damage to relevant authorities. The *Archaeological Practice and Heritage Protection in the Kurdistan Region of Iraq* project has already begun this process by creating a new museum space in Kalar to explain archaeological practice to a wider local public as well as museum school education boxes to encourage children, and young visitors and a wider public to engage in and help protect their local cultural heritage [11]. Since the end of the project, these resources have continued to be used widely and frequently.

#### Specific and immediate actions to mitigate future damage

A number of specific and immediate actions can also be taken to mitigate future damage to archaeological sites. First, as agricultural damage is the most prevalent threat, it is our recommendation that local farmers are made aware of the locations of archaeological sites as well as their generally low-quality soils and limited potential for cultivation. Local archaeologists and heritage professionals can then work together to develop alternative strategies with farmers to avoid or minimise damage to archaeological sites whenever possible, especially damage with heavy agricultural machinery. However, we do not recommend that site locations be widely publicised due to the threat of looting. Although we have stated that looting is minor compared to agricultural damage, it is still an ongoing threat and a challenge for antiquities officials to manage. Second, we recommend systematically communicating site information to municipal and regional planning authorities so that construction efforts may be directed away from sites wherever possible, or antiquities officials are able to record and investigate sites before construction begins or continues. Archaeologists from the Garmian Department of Antiquities have already signposted many prominent archaeological sites and have conducted a number of salvage excavations in the city of Kalar. Third and relatedly, sites can be protected from vehicular and natural erosion by (1) putting measures in place to prevent further natural erosion damage at the largest archaeological mounds; and (2) unless absolutely necessary, prohibiting vehicles from driving on top of archaeological sites or directing traffic along a single path to avoid continued damage.

Fourth and finally, our monitoring work has shown that few archaeological sites have been damaged by military conflict or looting in the last decade. The majority of conflict damage in particular appears to have occurred in the course of the 1980s Iran-Iraq War. However, the region is still subject to re-occurring conflicts and it is our recommendation that plans are put in place for the protection of archaeological sites and objects during times of conflict.

### Developing a common heritage policy for long-term damage mitigation

Finally, in order to implement these recommendations and ensure a sustainable and embedded programme of cultural resource management, Garmian archaeologists are liaising with local government, municipal planning departments, industries, and local communities to co-develop a common heritage policy. A common heritage policy ensures the protection of archaeological sites, outlines reporting mechanisms for damage and risk, as well as minimum requirements for archaeological assessment and recording to be carried out in advance of construction projects and other activities leading to site damage or destruction. This is work that is in progress as part of the larger *Archaeological Practice and Heritage Protection in the Kurdistan Region of Iraq* project and will be presented in the final and comprehensive report of project activities [11].

## Conclusions

Between 2018 and 2020, the A*rchaeological Practice and Heritage Protection in the Kurdistan Region of Iraq* project provided training and supported the work of Kurdish Iraqi archaeologists to record and monitor damage at all known archaeological sites in the Sirwan/Upper Diyala River Valley and adjacent landscapes. Our analysis demonstrates that the vast majority of known sites (86.7%) and more than a third of the archaeological area (38.6%, 278.9 ha) in the region have sustained one or more types of damage, calling for urgent action to develop an equitable and sustainable cultural heritage management plan that protects key archaeological sites and minimizes future damage. The greatest threat to archaeological sites in the region comes from industrial agricultural practices and construction activities. Damage from conflict and looting is comparatively less dramatic but must still be addressed. In order to ensure the protection of these sites in the future, we recommend a series of public information and engagement measures, the creation of an inter-institutional and community-supported heritage policy, as well as continued, locally-driven skills transfer and training.

## Supporting information

S1 Checklist Inclusivity in global research checklist. (PDF)

S1 Table Damage categories and sub-types used in site damage assessments (English and Kurdish). (PDF)

S2 Table Database of archaeological site damage assessments (2018–2020). (XLSX)
