# Supplementary material for: Embedding the remote sensing monitoring of archaeological site damage at the local level: Results from the “Archaeological practice and heritage protection in the Kurdistan Region of Iraq” project
Source: PLoS One. 2022 Jun 15;17(6):e0269796. doi: 10.1371/journal.pone.0269796 (PMC9200297; doi:10.1371/journal.pone.0269796)
Supplement: S1 Table — (PDF) [file pone.0269796.s002.pdf]

S1 Table. Damage categories and sub-types used in site damage assessments (English and Kurdish).

Damage Category	Damage Subtypes	Kurdish Translation
**Agriculture**<br>کشتوکاڵ	None<br>Channel Irrigation<br>Grazing/Trampling<br>Orchard<br>Ploughing<br>Terracing	هیچ (ئەگەر هیج زیانێک نەبوو)<br>جۆگەی ئاودێری<br>شوێنی ئاژەڵ_ ئەگەر شوێنەکە کرابوو بە شوێنی ئاژەڵ<br>باخ<br>کێڵاندن<br>شوێنی بە پلیکان کراو یان چین چین کراو
**Conflict**<br>شەڕو پێکدادان	Deliberate Vandalism<br>Explosives<br>Military Activity (e.g., re-use of ancient structures)	تێکدانی بە ئەنقەست _وەک نوسین و روشاندنی شوێنەوارەکان<br>تەقاندنەوە<br>چالاکی سەربازی
**Construction**<br>بنیاتنانەوە	Bulldozing (earthmoving)<br>Construction (e.g., buildings)<br>Dam Building (flood waters)<br>Mining/Quarrying<br>Modern Cemetery<br>Pipelines (e.g., power lines)<br>Refugee Encampment<br>Road Building<br>Vehicle Damage	ڕامالّین یان ڕوخاندن<br>بنیاتنانەوە _ (وەکو دروستکردنی بینا)<br>دروستکردنی بەنداو (ئاوی لافاو)<br>کانزاکاری _ کانە بەرد<br>گۆڕستانی نوێ<br>هێڵی بۆری _ئاو _نەوت<br>کەمپی پەنابەر<br>درەستکردنی ڕێگا<br>زیان بەهۆی ئامیرو ئامراز _وەکو ڕێگا خاکی و لاوەکیەکن لەسەر شوێنەوارەکان
**Natural Erosion**<br>ڕۆچون یان داخورانی سروشتی	River Erosion<br>Other Erosion	ڕۆچون_ڕامالّینی ڕوبار<br>رۆچون یان هەر جورە داخورانێکی تر
**Looting (presence and severity)**<br>هەبونی چاڵی تاڵانکردن و ڕادەی دژواری	Minor<br>Moderate<br>Severe	چاڵەکانی تاڵانکردن لاوەکی یە یان کەمە<br>چاڵەکانی تاڵانکردن مام ناوەندە (نە زۆرە_ نە کەمە)<br>چاڵەکانی تاڵانکردن بە زۆرە _بە سەختی ڕووبەڕوی تاڵانکردن بوەتەوە
